# Affective Reactions When Learning That Our Answer Is Biased: The Role of Negative Feedback in the Arousal of Epistemic Emotions

**DOI:** 10.5964/ejop.13847

**Published:** 2025-05-28

**Authors:** Katerina Nerantzaki, Paraskevi Stergiadou, Panayiota Metallidou

**Affiliations:** 1 Department of Psychology, Aristotle University of Thessaloniki, Thessaloniki, Greece; Lancaster University, Lancaster, United Kingdom

**Keywords:** heuristic biases, feedback, confusion, curiosity, epistemic emotions, surprise

## Abstract

This study investigated how different types of feedback influence emotional reactions in decision-making tasks involving high-confidence errors. The sample consisted of 596 undergraduate and postgraduate university students. Participants completed tasks and received either low informative feedback (indicating correctness) or high informative feedback (offering detailed explanations of correct answers). They reported their confidence levels and epistemic emotions of surprise, confusion, and curiosity. Participants reported epistemic emotions after each type of feedback. The results showed that confidence ratings did not differ between correct and incorrect answers. Incorrect answers elicited higher levels of surprise, curiosity, and confusion than correct answers. High informative feedback significantly reduced epistemic emotions, especially confusion, compared to low informative feedback. These results highlight the importance of detailed feedback in shaping epistemic emotions and enhancing learning in problem-solving contexts. Implications for research and teaching practices are discussed.

How do we feel when we are confident that we have answered a task correctly but are told that the answer is wrong? This study attempts to provide empirical evidence for affective reactions to different types of feedback received after providing a biased heuristic solution. Two different research traditions are combined, namely, the heuristics and biases tradition in decision-making situations (Kahneman, 2011) and the epistemic emotions tradition in learning situations (Muis et al., 2018; Vogl et al., 2020). Well-known decision-making tasks that tend to activate biased heuristic solutions were used as epistemic emotion-eliciting situations. Heuristic solutions are often accompanied by strong confidence in the correctness of an incorrect answer. Two types of external feedback were provided to inform participants about the correctness of their answers. The following subsections present information about heuristics and epistemic emotions in problem-solving situations.

## Heuristics

People are not always rational when they have to choose an answer or make a decision. Rather, they tend to use an effort-reducing method of processing information called heuristics (see also Kahneman & Tversky, 2013). The term “heuristics” is derived from the Greek word “evrisko”, which means to find or to discover. Heuristics are widely used in decision-making problems because they help to speed up the decision-making or problem-solving processes (Emiliano, 2015). According to Shah and Oppenheimer (2008), heuristics facilitate effort reduction by, (a) considering fewer cues, (b) reducing the effort to retrieve cue values, (c) reducing the weighting of cues, (d) combining less information, and (e) exploring fewer alternatives.

To illustrate, a heuristic method is a quick way to solve a problem with a solution that is not the best but the most likely one, like a “mental shortcut” (Kahneman, 2011). This has the advantage of saving time and effort while simultaneously ending up (very often) making the right decision in the end. However, the disadvantage of these mental shortcuts and effort-reducing solutions is that they can sometimes lead to errors and biased judgments or logical fallacies (Liberali et al., 2012). They are statements that seem to be logical, but when examined using the rules of logic, turn out to be biased. Logical fallacies are often associated with a high feeling of confidence in an incorrect answer (Kahneman & Tversky, 2013). Metacognitive feeling of confidence has been found to be associated with affective reactions (Nerantzaki & Efklides, 2019; Vogl et al., 2019).

In general, many theorists have acknowledged the important role of affect in decision-making processes (Garcés & Finkel, 2019). For instance, the “affect heuristic” (Slovic et al., 2007) refers to a mental shortcut in which individuals make quick decisions primarily guided by their emotions. While the important role of affective processes in the formulation of judgments or decision making is well established (Garcés & Finkel, 2019; Schwarz & Clore, 2007), little is known about affective responses following the recognition of biases in our judgments. The present study aims to fill this gap by providing empirical evidence on affective reactions following biased heuristic errors. One category of affective reactions that is closely related to problem-solving activities (D’Mello & Graesser, 2014) and arises from unexpected and complex information (Chevrier et al., 2019; Vogl et al., 2019) are the so-called epistemic emotions.

## Epistemic Emotions

*Epistemic emotions* were initially described by philosophers as affective states that can motivate critical reflection, knowledge acquisition, and exploration (see Brun et al., 2008; Morton, 2010). From a philosophical perspective, epistemic emotions drive knowledge acquisition and knowledge inquiry (Muis et al., 2018). From an educational psychology perspective, epistemic emotions are categorized within the broader domain of academic emotions (Pekrun, 2024). Academic emotions, which are specific to educational contexts, are closely associated with learning processes and academic achievement (Pekrun, 2006, 2016). Pekrun and Stephens (2012) defined epistemic emotions as emotions related to knowledge and knowledge-generating activities. Any emotion associated with knowledge can be characterized as epistemic (Morton, 2010; Muis et al., 2018). However, as Brun et al. (2008) suggest, curiosity, surprise, and confusion are epistemic emotions per se, whereas other affective states can fall into different emotional categories depending on their object focus. For instance, frustration or anxiety, arising from the inability to solve a mathematics problem, may be considered epistemic emotions if they stem from cognitive incongruity and the unresolved problem itself. However, if they are primarily focused on personal failure and the perceived inability to arrive at the correct solution, they should be classified as achievement emotions (Muis et al., 2015).

Specifically, epistemic emotions are triggered by a lack of prior knowledge, conflicting information, or a mismatch between new information and existing knowledge schemas (Muis et al., 2021; Nerantzaki et al., 2021; Vogl et al., 2019). A recent analysis of think-aloud protocols identified three key factors that precede epistemic emotions: assessments of epistemic (in)congruence (the congruence or incongruence between prior knowledge and new information), assessments of the novelty or conflicting information, and judgments about the achievement of epistemic goals (Chevrier et al., 2019). Within this context, it is important to distinguish between cognitive conflict and schema incongruence, as both concepts are closely related to epistemic emotions. *Cognitive conflict* arises when learners encounter contradictory information, in tasks involving conflicting responses or in texts presenting opposing perspectives on a topic (D’Mello & Graesser, 2014; Nerantzaki et al., 2024). *Schema incongruence* occurs when new information does not align with existing cognitive frameworks, prompting cognitive adjustments to resolve the mismatch (Reisenzein et al., 2019).

Schema incongruence also occurs when task-related feedback reveals that one’s beliefs are incorrect, such as in “high-confidence errors” (Marshall & Brown, 2006; Vogl et al., 2019). “High-confidence errors” refer to incorrect responses that individuals strongly believe to be correct. Vogl and collaborators (2019, 2020) examined those errors in the context of feedback provided after trivia questions were answered incorrectly. Their findings support propositions that the mismatch between expected and actual outcomes is a prime driver of epistemic emotions. This phenomenon can be further explained through the concept of cognitive dissonance. Cognitive dissonance refers to the psychological discomfort that arises when newly acquired information contradicts prior beliefs, expectations, or knowledge (Festinger, 1957). This tension creates a motivational drive to resolve the inconsistency, by adjusting one’s beliefs, seeking additional information, or reinterpreting the conflicting evidence (Harmon-Jones & Mills, 2019). In the context of negative feedback, this process can trigger epistemic emotions such as confusion, surprise, or curiosity as individuals attempt to reconcile the discrepancy and restore cognitive consistency (Stergiadou et al., 2023; Vogl et al., 2020).

Despite the similarities, it has been proposed that surprise, confusion and curiosity are triggered by different cognitive states (Nerantzaki et al., 2021). *Surprise* is a leading epistemic emotion that occurs when a stimulus is unexpected, discrepant, or inconsistent with a previously activated cognitive schema (Reisenzein et al., 2019; Topolinski & Strack, 2015). *Confusion* occurs when people encounter information that they cannot easily understand. It is generally associated with challenging tasks in the presence of impasse, complexity, conflict, contradiction, or incongruity (D’Mello & Graesser, 2012; Silvia, 2009). *Curiosity* represents a driving force or desire to seek new information that motivates the exploration and acquisition of knowledge (Kashdan & Silvia, 2009). Curiosity is aroused by unexpected events, unsolved problems, ambiguous ideas, conceptual puzzles, or information that makes one aware of gaps in one’s knowledge. It is a complex emotion, and its arousal involves both positive feelings (e.g., the satisfaction of learning something new, and negative feelings (e.g., impatience due to lack of knowledge) (Ainley, 2019).

In summary, the role of epistemic emotions in problem-solving activities is only beginning to be explored. A recent study examined the role of external feedback and found that the intensity of these emotions increased in proportion to the confidence participants had in their incorrect answers (Vogl et al., 2019). The focus of the present study was to extend these findings by examining how different types of feedback influence epistemic emotions in decision-making tasks involving high-confidence errors. For this purpose, two widely recognized scenarios were used. The first scenario was the conjunction fallacy, where participants were asked to estimate the probability of two events occurring together, often resulting in an overestimation of the combined probability (Liberali et al., 2012). The second scenario is a cognitive reflection task that tests participants' ability to override an intuitive response (Brañas-Garza et al., 2019). The choice of these scenarios was deliberate. The scenarios are not inherently difficult when approached with logical reasoning. However, many people tend to give incorrect answers, mainly due to an overestimation of their own intuitive judgement (Kahneman, 2011). We assumed that these scenarios would induce a high level of confidence in the participants’ answers, even though these answers ultimately were incorrect. In line with this, both scenarios are anticipated to evoke epistemic emotions, as they involve the evaluation of epistemic incongruence (Chevrier et al., 2019; Vogl et al., 2019).

## The Present Study

The present study aimed to investigate epistemic emotions (surprise, confusion, curiosity) following different types of feedback after participants engaged in a faulty heuristic solution. This work bridges two different research traditions: the heuristics and biases tradition, which focuses on decision-making under uncertainty (Kahneman & Tversky, 2013) and the epistemic emotions tradition in learning and decision-making situations (Muis et al., 2015; Vogl et al., 2019). While prior research has explored epistemic emotions in response to schema-discrepant information (e.g., Chevrier et al., 2019; Muis et al., 2015; Vogl et al., 2019), these emotions have not been specifically examined in the context of heuristic errors that generate logical inconsistencies.

To address this gap, we provided participants with two types of feedback: “low-informative” feedback that merely indicated whether an answer was correct and “high-informative” feedback that included detailed justifications for the correct response. We hypothesized that high-informative feedback would facilitate the integration of new information into existing cognitive schemas (Muis et al., 2015), potentially reducing the intensity of epistemic emotions as cognitive dissonance is resolved (Nerantzaki et al., 2021).

Based on these considerations, two hypotheses were proposed:

1. Performance would be negatively related to epistemic emotions. Specifically, surprise, curiosity, and confusion were expected to arise when discrepant information challenged existing cognitive schemas (*Hypothesis 1a*). The reported epistemic emotions were expected to be higher following biased heuristic answers compared to correct answers (*Hypothesis 1b*).

2. A significant decrease in the intensity of epistemic emotions was expected in the second measurement, following the provision of detailed feedback that explained the correct answer’s underlying principles (*Hypothesis 2*).

## Method

### Participants

The sample consisted of 596 (66.4% female) undergraduate and postgraduate students (*M*_age_ = 25.09, *SD* = 6.84) from Greek university departments. To broaden generalizability of the results, participants were included students from various departments: psychology (*n* = 80), engineering (*n* = 76), natural sciences (*n* = 158), education (*n* = 25), economics (*n* = 25), law school (*n* = 10), and other departments. To enhance the generalizability of the findings to a broader student population, the sample included students from a diverse range of academic disciplines, including psychology, engineering, natural sciences, education, economics, law, and other fields. Participants completed the study online and were recruited through a university-related Facebook page. Their participation was voluntary.

### Materials

#### Main Task

The main task involved two well-known decision-making scenarios. Many people give incorrect answers to these problems because they use a faulty procedure.

(a) The Job Scenario, also known as the “Linda problem” (Tversky & Kahneman, 1973), is designed to trigger the representativeness heuristic. The representativeness heuristic occurs when people assume that specific details are more likely than a general description (Richie & Josephson, 2018). In the Linda problem, participants are given a description of a woman who is 31 years old, very intelligent, and politically active, particularly in social justice issues. They are then asked to judge the likelihood of different statements about her. The “conjunction fallacy” demonstrated by the Linda problem, occurs when people mistakenly believe that the probability of two or more events occurring together is higher than the probability of any one of those events occurring alone (Kahneman, 2011). This violates basic principles of probability theory because the likelihood of two events occurring together (A and B) can never be greater than the likelihood of either event occurring alone (A or B).

(b) The Disease Scenario is a cognitive reflection task designed to measure how well people can override an initial, incorrect response (Toplak et al., 2011). It was first introduced by Kahneman and Frederick (2002) as a test of common reasoning errors, where people quickly give the first answer that comes to mind without thinking carefully enough to spot their mistake. In this scenario, participants are presented with a situation in which a doctor develops a new drug to treat a disease. The doctor tests the drug on a group of patients and compares their results with a control group that doesn't receive the treatment. The task usually includes a table showing the success or failure of the treatment, which participants use to evaluate statistical data and make probability judgements. When solving this problem, many people make a typical reasoning error: they behave like “cognitive misers” (Toplak et al., 2014). To solve the problem correctly, participants need to critically reassess their initial answer and engage in more thoughtful reasoning to arrive at the correct solution (Frederick, 2005).

#### Performance and Epistemic Emotions Measurement

Performance was estimated as correct (1) or wrong (0) for each scenario. Participants were asked to choose the answer they thought was correct and to report how difficult it was to find the answer in each scenario and how confident they were about their answer on a 4-point Likert scale (1 = *Not at all* to 4 = *Very much*). Then, to elicit the target emotions, they received feedback indicating the correct answers and were asked to rate how surprised, curious, and confused they felt at that very moment on a 4-point Likert scale (1 = *Not at all* to 4 = *Very much*).

After the first measurement of their epistemic emotions, they again received “high informative” feedback, in which the correct answer was explained in detail. A second measurement of epistemic emotions was then administered. To avoid the effects of time, half of the participants were randomly assigned to receive the “high informative” feedback immediately after the completion of the first epistemic emotions measurement (Condition A). The other half received it after the completion of the first measurement of epistemic emotions in both tasks (Condition B). No statistically significant differences were found in epistemic emotions (*ps >* .05) or performance (*ps >* .05) between the two conditions. The design of the study is outlined in Schema 1 below.

##### Schema 1

###### The Design of the Study

**Phase Α’**
- Reading scenario.
- Selection of the correct answer.
- Measurement of the feeling of difficulty and confidence for each answer.

**Phase Β’**
- Feedback about the correct answer after the feeling of confidence in each scenario (low informative feedback).
- 1st measurement of epistemic emotions (surprise, curiosity and confusion) after the first feedback.

**Phase C’**
- Condition 1 (feedback after the completion of each task).
  - Feedback with detailed justification of the correct answer (high informative feedback).
  - 2nd measurement of epistemic emotions (surprise, curiosity and confusion) after the second feedback.
- Condition 2 (feedback after the completion of both tasks).
  - Feedback with detailed justification of the correct answer after the first measurement of epistemic emotions in both tasks (high informative feedback).
  - 2nd measurement of epistemic emotions (surprise, curiosity and confusion) after the second feedback.

### Procedure

The research was conducted using the online open-source platform “LimeSurvey”. The participation was voluntary and anonymous. No sensitive personal information was asked. The participants reported their gender, level of studies, and university department. The study also was fully in line with rules of ethics of the American Psychological Association and with the European Union Regulation on sensitive personal data (GDPR; https://gdpr.eu/tag/gdpr/), as in force from 25 May 2018 and as applicable in the Greece according to Law 4624/2019 (Issue A´ 137/29.08.2019).

It is important to note that scenarios were translated by the researchers and were provided in Greek language (see the Appendix). The tasks were translated using a rigorous forward-backward translation process (see Muis et al., 2015), followed by a review by a bilingual expert to ensure accuracy. Additionally, the scenarios were pilot tested with participants from the target population to identify and address any potential cultural misinterpretations that may have been missed in the translation process (Harkness et al., 2010). The results showed that the success rates (~ 40%) in the translated scenarios were consistent with those in the original scenarios.

Participants were first informed of the purpose of the study, the profile of the sample, and the confidentiality procedures. When the data were coded, each participant was given a corresponding number as a name, from 1 to 596. They were also informed that they could withdraw from the survey at any time they wished. The timing option was also activated to determine the time spent by the participants in each scenario to exclude either random answers (too short time spent in each task) or answers that might result from searching on the Internet or giving too much thought (too much time spent). The time allocated for each task was determined based on a pilot study with 30 participants. This timing was influenced by multiple factors, including the number of words in each scenario (i.e., scenario length), the difficulty of the task, and the available response options. Using the average engagement times observed in the pilot study, standardized time limits were established for each scenario. Participants who spent significantly more or less time on a task- relative to the scenario’s average time- were excluded to minimize the likelihood of random responses. More especially, forty (40) participants were excluded from the initial sample (*n* = 636) due to either very short (-3 *SD*) or very long time spent (+3 *SD*). The response time in the Job scenario was *M* = 77 sec. and in the Disease scenario was *M* = 65 sec. The data together with a data dictionary, the analysis script and the study materials are available in full at Nerantzaki et al. (2025).

## Results

The percentage of correct answers and biased heuristic answers was calculated for each task to create two groups: those who found the correct answer and those who did not. Descriptive statistics for all the variables in each scenario are presented in Table 1. Specifically, in the *Job Scenario*, 241 (40.5%) of the participants chose the correct answer. In the *Disease Scenario*, 279 (46.7%) participants selected the correct response.

**Table 1:** Descriptive Statistics of Variables in Each Scenario

	Correct Answer		Incorrect Answer (heuristic)	
Variable	*Mean*	*SD*	*Mean*	*SD*
Job Scenario				
Surprise^a^	1.54	0.65	2.32	0.96
Confusion^a^	1.27	0.56	2.17	0.97
Curiosity^a^	1.68	0.88	2.50	0.98
Surprise^b^	1.37	0.64	1.99	0.92
Confusion^b^	1.15	0.47	1.45	0.75
Curiosity^b^	1.54	0.78	2.04	0.89
Disease Scenario				
Surprise^a^	1.44	0.62	2.47	0.96
Confusion^a^	1.17	0.43	2.59	0.95
Curiosity^a^	1.64	0.81	2.66	0.95
Surprise^b^	1.19	0.48	2.09	0.95
Confusion^b^	1.09	0.31	1.63	0.88
Curiosity^b^	1.39	0.71	2.03	0.95

^a^immediate “low informative” feedback. ^b^subsequent “high informative” feedback.

### Task Performance and Time of Measurement Effect

Pearson’s correlations were computed within and across each type of feedback for both scenarios to explore: (a) the interrelations between performance and epistemic emotions, and (b) the extent to which these relations differed between different types of feedback. Correlations ranged from *r* = - 0.22, *p* < .001 to *r* = 0.70, *p <* .001 (see Table 2).

**Table 2:** Pearson Correlations Between Epistemic Emotions and Performance

Job Scenario							
Variable	1	2	3	4	5	6	7
1. Performance	—						
2. Surprise^a^	-0.41**	—					
3. Confusion^a^	-0.47**	0.65**	—				
4. Curiosity^a^	-0.39**	0.58**	0.58**	—			
5. Surprise^b^	-0.35**	0.54**	0.47**	0.40**	—		
6. Confusion^b^	-0.22**	0.30**	0.35**	0.24**	0.44**	—	
7. Curiosity^b^	-0.28**	0.39**	0.36**	0.47**	0.56**	0.38**	—
Disease Scenario							
Variable	1	2	3	4	5	6	7
1. Performance	—						
2. Surprise^a^	-0.53**	—					
3. Confusion^a^	-0.68**	0.70**	—				
4. Curiosity^a^	-0.50**	0.67**	0.65**	—			
5. Surprise^b^	-0.41**	0.57**	0.60**	0.57**	—		
6. Confusion^b^	-0.37**	0.39**	0.49**	0.37**	0.53**	—	
7. Curiosity^b^	-0.35**	0.44**	0.44**	0.51**	0.68**	0.43**	—

^a^epistemic emotions after the “low informative”. ^b^epistemic emotions after the “high informative” feedback.

**p < .001.

As expected, performance in both scenarios was negatively associated with epistemic emotions. Correlations were stronger with epistemic emotions following the first feedback, particularly with surprise and confusion, and lower with curiosity. Performance was also correlated with epistemic emotions after the second feedback, although these correlations were generally medium to low, with surprise showing a slightly higher correlation. There was also a complex network of relationships between epistemic emotions across scenarios. The emotions following the first feedback were all interrelated, with the strongest correlations found between surprise and confusion. This pattern of correlations was maintained at the second measurement time, with the highest r between surprise and curiosity in both scenarios. Furthermore, most of the epistemic emotions experienced after the first feedback were related to those after the second feedback, with surprise showing significant correlations with almost all the emotions observed after the second feedback. Finally, each epistemic emotion was significantly correlated with its counterpart across the two measurements.

In addition, a series of 2 (performance -correct vs incorrect) by 2 (type of feedback -low vs high informative feedback) within-subjects’ ANOVAs were conducted for each epistemic emotion in each scenario. To ensure the validity of the ANOVA results, the assumptions of sphericity and homogeneity of variances were tested. Mauchly’s test of sphericity was not significant (*ps >* .05), indicating that the assumption of sphericity was met for each epistemic emotion in each scenario. Furthermore, Levene’s test of equality was also not significant (*ps >* .05). The main results are presented in Table 3.

**Table 3:** Performance Effect and Feedback Type Effects on Epistemic Emotions in Two Scenarios

Variable	Effect of Feedback	Effect of Performance	Interaction
Job Scenario			
Surprise	*F*(1, 593) = 46.87, *p* < .001, $\eta_{p}^{2}$ = .07	*F*(1, 593) = 139.53, *p* < .001, $\eta_{p}^{2}$ = .19	*F*(1, 593) = 5.38, *p* = .021, $\eta_{p}^{2}$ = .01
Confusion	*F*(1, 593) = 127.63, *p* < .001, $\eta_{p}^{2}$ = .18	*F*(1, 593) = 144.52, *p* < .001, $\eta_{p}^{2}$ = .20	*F*(1, 593) = 65.01, *p* < .001, $\eta_{p}^{2}$ = .10
Curiosity	*F*(1, 593) = 53.83, *p* < .001, $\eta_{p}^{2}$ = .08	*F*(1, 593) = 109.50, *p* < .001, $\eta_{p}^{2}$ = .16	*F*(1, 593) = 15.66, *p* < .001, $\eta_{p}^{2}$ = .03
Disease Scenario			
Surprise	*F*(1, 593) = 77.87, *p* < .001, $\eta_{p}^{2}$ = .12	*F*(1, 593) = 308.52, *p* < .001, $\eta_{p}^{2}$ = .34	*F*(1, 593) = 2.79, *p* = .095, $\eta_{p}^{2}$ = .01
Confusion	*F*(1, 593) = 238.91, *p* < .001, $\eta_{p}^{2}$ = .29	*F*(1, 593) = 411.05, *p* < .001, $\eta_{p}^{2}$ = .41	*F*(1, 593) = 171.62, *p* < .001, $\eta_{p}^{2}$ = .22
Curiosity	*F*(1, 593) = 126.61, *p* < .001, $\eta_{p}^{2}$ = .17	*F*(1, 593) = 190.29, *p* < .001, $\eta_{p}^{2}$ = .24	*F*(1, 593) = 23.40, *p* < .001, $\eta_{p}^{2}$ = .04

**Figure 1:**
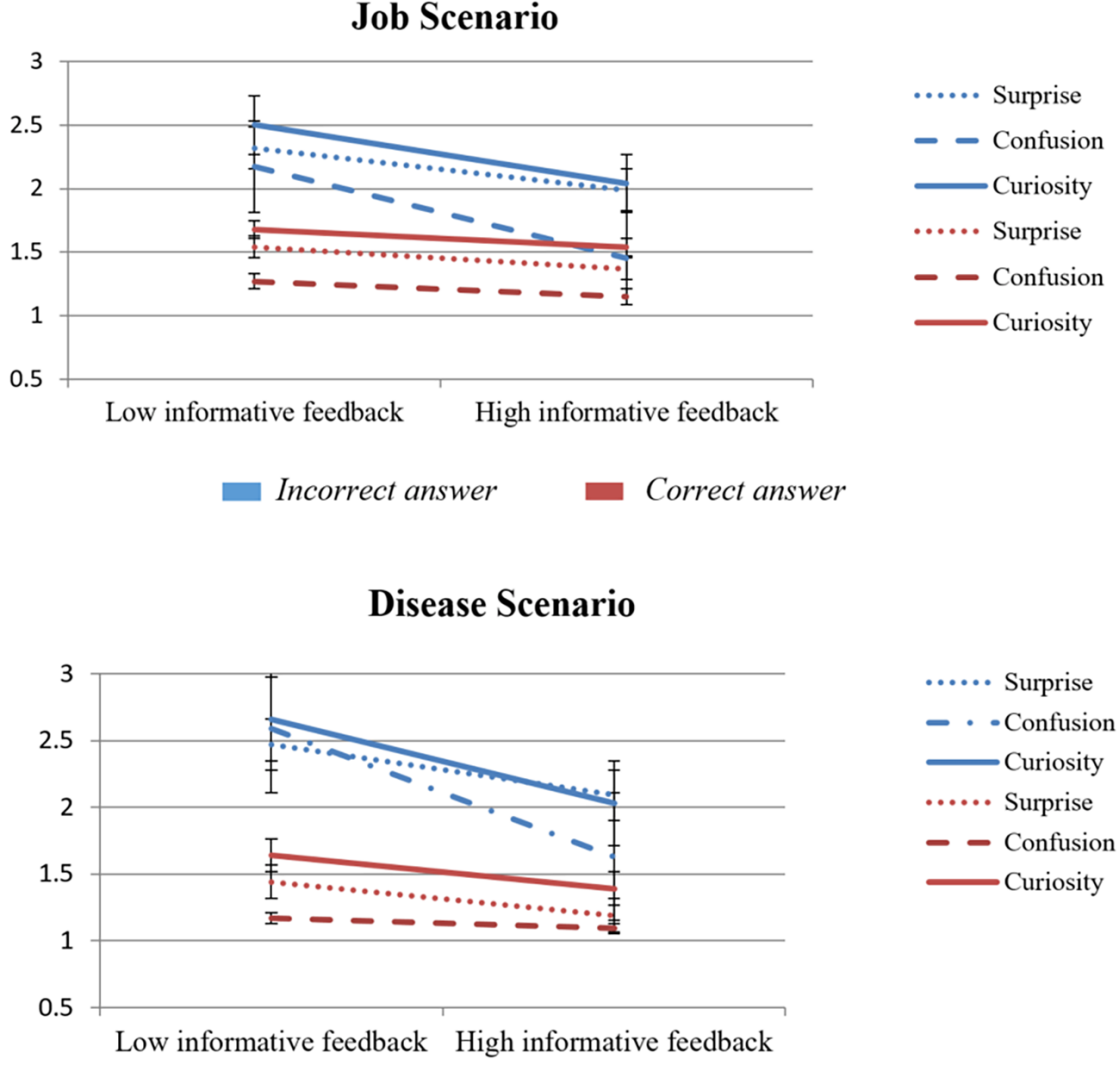
Plotted Change in Epistemic Emotions over the Scenarios

The main effect of feedback type was found to be significant in all cases (*p <* .001). As expected, the mean ratings of all epistemic emotions (surprise, confusion, curiosity) were significantly higher in the first measurement (low informative feedback) compared to the second one (high informative feedback). The main effect of performance was also found to be significant in all cases (see Table 3). To illustrate, epistemic emotions were statistically significantly higher (*p <* .001) for participants who chose the biased heuristic answer compared to participants who chose the correct answer. Confusion was the only emotion that showed the most drastic decrease in the second measurement but only for those who chose the biased heuristic answer (see Figure 1).

Additionally, it is important to consider the effect sizes of the results. Based on the guidelines provided by Cohen et al. (2003), $\eta_{p}^{2}$ ≈ .01 indicates a small effect, $\eta_{p}^{2}$ ≈ .06 represents a medium effect, and $\eta_{p}^{2}$ ≈ .14 reflects a large effect size. For the emotion of surprise, the performance effect showed a large effect size, suggesting that performance significantly influenced participants' feelings of surprise. The feedback effect, on the other hand, showed a medium effect size, suggesting that the feedback type (low vs. high informative) had a moderate effect on surprise ratings. This pattern was consistent across both scenarios, reinforcing the robust role of performance in eliciting surprise. Curiosity followed a similar pattern. The performance effect again had a large effect size, showing that participants who performed better (correct answers) experienced significantly higher levels of curiosity. However, the feedback effect was medium in size, suggesting a more moderate influence of feedback type on curiosity, which was consistent across both scenarios. For confusion, both the performance and feedback effects were large, with $\eta_{p}^{2}$ values approaching or exceeding .14. This highlights the strong role that both performance (correct vs. biased answer) and feedback type (low vs. high informative) play in eliciting the emotion of confusion. In particular, the large effect of feedback underscores its critical role in eliciting confusion.

Furthermore, the interaction (type of feedback x performance) was statistically significant in all cases (*p* < .05), except for the emotion of surprise in the second scenario (*p* = .09). The interaction effect indicates that the effect of feedback type depends on the performance variable. More specifically, the variation in epistemic emotions between the two types of feedback was higher for those who chose the biased heuristic answer than for those who found the correct one. However, the effect sizes of the interaction were small for surprise and curiosity in both tasks ($\eta_{p}^{2}$ < .04). A significantly larger interaction effect was found for the emotion of confusion ($\eta_{p}^{2}$ = .10 for the Job Scenario and $\eta_{p}^{2}$ = .22 for the Disease Scenario).

## Discussion

The present study investigated how different types of feedback influence emotional reactions in decision-making tasks involving high-confidence errors. Participants completed two decision-making scenarios that activate biased heuristic solutions and received either low informative feedback (indicating correctness) or high informative feedback (offering detailed explanations of correct answers). They reported their confidence levels and epistemic emotions of surprise, confusion, and curiosity. Participants’ performance and the type of external feedback on their performance significantly affected all epistemic emotions. The results confirm previous evidence for the important role of high-confidence errors in eliciting epistemic emotions but also extend the role of external feedback. To illustrate, negative external feedback following an incorrect answer led to significantly higher levels of surprise, confusion, and curiosity in both tasks compared to those emotions following the correct answers.

Epistemic emotions were also negatively associated with performance in both tasks. The findings are consistent with the notion that cognitive incongruity serves as a key trigger for epistemic emotions (D’Mello & Graesser, 2012; Vogl et al., 2019). It seems that an incongruity between an activated schema (response) and newly received information (feedback) activated epistemic emotions. Participants were forced to construct a new schema by assimilating new information (Efklides, 2017). The aroused epistemic emotions reflect the activation of such revision processes (Vogl et al., 2019). Correlations between performance and epistemic emotions were stronger following the first feedback, as the second feedback provided all the necessary information to finalize the accommodation of the new schema. In contrast, participants who answered correctly experienced feedback that aligned with their existing cognitive schemas, resulting in low-intensity epistemic emotions from the outset.

Furthermore, one of the most interesting findings of the present study was the significant decrease in epistemic emotions after receiving highly informative feedback. The first external feedback activated epistemic emotions and induced a cognitive imbalance (Muis et al., 2015). Indeed, the intensity of these emotions was shaped by participants’ confidence in their incorrect answers, indicating a mismatch between their prior beliefs and the correct answer (Vogl et al., 2019). The cognitive imbalance was restored after the second feedback, which provided all the explanations necessary to assimilate the new information into one's existing cognitive schemas (Nerantzaki et al., 2021). Vogl et al. (2019) proposed that epistemic emotions are inherently situation-dependent, changing over time in response to variations in task information. This is indicative of the dynamic nature of epistemic emotions in learning or decision-making situations (Nerantzaki, et al., 2024; Pekrun, 2024).

However, there were also some differences in the arousal of epistemic emotions. Although all epistemic emotions followed a similar pattern of activation, the feedback effect was lower for curiosity and surprise, and higher for confusion. The differences in effect sizes for surprise, curiosity, and confusion can be interpreted through the distinct cognitive mechanisms underlying each epistemic emotion (Nerantzaki et al., 2021; Vogl et al., 2021). Surprise is often triggered by unexpected outcomes or violations of expectations (Reisenzein et al., 2019). Curiosity arises from an intrinsic desire for knowledge and information-seeking behavior, often stimulated by uncertainty or gaps in understanding (Litman, 2008). Both emotions strongly depend on cognitive evaluations of performance. In contrast, confusion demonstrated a higher effect size for feedback, but also of performance, indicating a stronger link with difficulty in processing information (D’Mello et al., 2014; Nerantzaki et al., 2024). Confusion arises during complex learning activities that involve challenging tasks, particularly when encountering discrepancies that disrupts understanding (D’Mello & Graesser, 2014). That means that the nature of the task plays a crucial role in determining which epistemic emotions are elicited (Vogl et al., 2021).

In addition, confusion was the emotion that showed the most drastic decrease in the second measurement for those who chose the biased heuristic answer. Informative feedback seems to be important in reducing confusion caused by information that is incongruent with previous beliefs. Confusion is triggered by complex information that is not fluently understood (D’Mello & Graesser, 2012; Silvia, 2009). Unlike surprise, which primarily refers to unexpected events (Reisenzein et al., 2019), confusion can also occur under so-called “mismatch conditions”, such as incongruence, dissonance, or conflict (D’Mello et al., 2014). In fact, confusion is not simply the detection of a mismatch, but an intense emotional state characterized by significant cognitive conflict, leading to uncertainty about how to proceed (D’Mello et al., 2014). Furthermore, D’Mello and Graesser (2014) suggested that confusion persists until the cognitive conflict is effectively resolved.

Overall, decision-making processes are often influenced by affective states (Garcés & Finkel, 2019). Our findings provide support for the proposition of Vogl et al. (2019) that epistemic emotions arise during high confidence errors, but we also found that the type of external feedback matters. The first negative feedback activated epistemic emotions and cognitive imbalance, which were restored after the second feedback, which presented the logical principle underlying the correct answer. In what follows, we consider limitations and directions for future work. We also discuss educational implications for research on epistemic emotions.

### Limitations

While the findings of this study are significant, several limitations should be acknowledged. First, the sample was exclusively composed of university students, which may limit the generalizability of the results to non-student populations or individuals outside of academic contexts. Expanding the sample to include a more diverse range of participants could enhance the applicability of the findings. Second, the study relied on a specific set of tasks and stimuli, which raises concerns about the robustness of the results. The findings need to be tested with varied types of stimuli to ensure they are not influenced by intrinsic characteristics of the specific scenarios used. Additionally, participants’ prior knowledge was not assessed. This structured approach may not fully capture the complexity of real-world decision-making processes. Third, individual differences such as linguistic abilities, learning styles, and cultural interpretations of scenarios were not examined. These factors could play a significant role in shaping epistemic emotions and their impact on performance. Future research should explore how task characteristics, such as the distinction between reading-based versus nonverbal problem-solving tasks, influence the arousal of epistemic emotions and their effect on learning outcomes. Investigating how individual differences and contextual factors contribute to variability in emotional responses could also provide deeper insights into the mechanisms underlying learning and decision-making processes. Finally, future research could explore whether similar patterns of epistemic emotions and cognitive processes emerge in decision-making contexts beyond probabilistic reasoning, thereby enhancing the generalizability of these results.

### Implications for Research and Practice

The findings emphasize the situational nature of epistemic emotions, which fluctuate in response to different types of external feedback. These emotions are inherently dynamic, evolving alongside the progression of cognitive processing (Efklides, 2017). This underscores the importance of employing repeated measures to track changes in epistemic and other affective states throughout problem-solving tasks and learning activities. In the context of problem-solving, the key question is not merely whether an epistemic emotion is elicited but how it evolves during cognitive engagement. For example, initial low levels of surprise may intensify into heightened surprise and curiosity upon detecting a discrepancy (see Vogl et al., 2019). Conversely, prolonged confusion may lead to negative affective states such as frustration or boredom (D’Mello & Graesser, 2014). Understanding these emotional dynamics is essential for designing interventions and feedback mechanisms that support effective learning and decision-making processes.

The present study underscored the importance of informative feedback. While feedback is widely acknowledged as a key factor in enhancing learning outcomes, its effectiveness can vary greatly depending on the context (Frank et al., 2018). Recent findings by Cáceres et al. (2021) highlight that elaborated feedback, which provides detailed explanations, is more effective than simple feedback that merely indicates the correct response. In the current study, participants who received detailed explanations were able to resolve their cognitive imbalances, suggesting that such feedback not only corrects errors but also supports the assimilation of new information into existing cognitive schemas. For instance, in domains like mathematics or logical reasoning, feedback that pinpoints specific reasoning errors and offers constructive alternatives has been shown to significantly improve problem-solving skills. These findings emphasize the importance of understanding the dynamic interplay between feedback and cognitive processes, shedding light on the complex mechanisms underlying effective problem-solving and learning.

The study highlights the pivotal role of external feedback in influencing epistemic emotions following a faulty heuristic solution. External feedback serves dual purposes: it can act as a trigger for epistemic emotions (Vogl et al., 2020), encouraging students to engage actively with educational material, or as a regulator of emotional responses in cases of cognitive dissonance or conflict. From an educational standpoint, these findings emphasize that feedback should be seen not merely as a tool for transmitting information but also as a mechanism for managing emotional responses. Teachers can enhance their practices by incorporating highly informative feedback that addresses cognitive imbalances and supports their resolution during learning.

However, as the effect sizes for the feedback effect on curiosity and surprise were lower (Cohen et al., 2003), it is important to consider their potential impact in educational settings. These effects may interact with other cognitive factors, such as task difficulty, motivation, and prior knowledge, potentially amplifying their influence in real-world learning environments. Future research could explore whether different types of feedback enhance these emotions more effectively, leading to stronger learning outcomes. Since curiosity and surprise are primarily driven by cognitive evaluations of performance, educators can foster these emotions by incorporating open-ended problems, inquiry-based learning, and scenarios that introduce uncertainty to stimulate exploration. On the other hand, confusion, which is closely tied to both feedback and performance, should be carefully managed to ensure it remains productive. Providing timely feedback, structured guidance, and scaffolding can help students navigate challenging tasks without becoming overwhelmed (D’Mello et al., 2014). By promptly and effectively addressing confusion, educators can mitigate the risk of students becoming overwhelmed or disengaged (D’Mello & Graesser, 2012; Silvia, 2009). This approach underscores the importance of integrating both cognitive and emotional dimensions into feedback strategies to optimize learning outcomes.

### Conclusion

In conclusion, this study contributes to the expanding literature on emotions by examining the pivotal role of external feedback. The findings build on prior research by highlighting the dual function of feedback: as a trigger for epistemic emotions (Vogl et al., 2019) and as a regulator that helps manage these emotions during cognitive processing. Given the critical role of epistemic emotions in driving knowledge exploration and promoting deeper learning (Pekrun, 2024), the study underscores the importance of designing feedback mechanisms that not only challenge learners but also support them in achieving resolution and understanding. The positive effects of curiosity on learning, alongside the potential drawbacks of prolonged high confusion, highlight the necessity of feedback as a tool for emotional regulation. These insights open avenues for future research to further explore how feedback can be optimized to balance challenge and support, ultimately enhancing learning outcomes.

## Supplementary Materials

For this article, the following Supplementary Materials are available:

- Data. (Nerantzaki et al., 2025)
- Code. (Nerantzaki et al., 2025)
- Codebook. (Nerantzaki et al., 2025)
- Study materials. (Nerantzaki et al., 2025)

## Data Availability

For this article, data, codebook, program codes, and translated survey items are available at Nerantzaki et al. (2025).

## Appendix

**Table:** Scenarios With English Translation

Scenario & Feedback: Original Language and Translation					
Job Scenario in Greek			Job Scenario in English		
Η Λίντα είναι 31 ετών, δεν έχει παντρευτεί, είναι πετυχημένη και πολύ έξυπνη. Το πτυχίο της ήταν στη Φιλοσοφική. Ως φοιτήτρια, ενδιαφερόταν πολύ για θέματα διακρίσεων και κοινωνικής δικαιοσύνης, και έπαιρνε μέρος σε αντιπυρηνικές διαδηλώσεις. Α. Η Λίντα είναι τραπεζική υπάλληλος. B. Η Λίντα είναι τραπεζική υπάλληλος και ενεργό μέλος του φεμινιστικού κινήματος. Γ. Η Λίντα είναι τραπεζική υπάλληλος, ενεργό μέλος του φεμινιστικού κινήματος και ασχολείται με τη γιόγκα			Linda is 31 years old, single, outspoken, and very bright. She majored in philosophy. As a student, she was deeply concerned with issues of discrimination and social justice and participated in anti-nuclear demonstrations. Α. Linda is a bank teller. Β. Linda is a bank teller and is active in the feminist movement. C. Linda is a bank teller and is active in the feminist movement and yoga instructor		
Feedback in Greek			Feedback in English		
1. Η σωστή απάντηση είναι το A. Η Λίντα είναι τραπεζική υπάλληλος 2. Η πιθανότητα της σύζευξης δύο συμβάντων ή και τριών δεν μπορεί να είναι πιο μεγάλη από την πιθανότητα του κάθε συμβάντος από μόνο του. Αποτελεί σφάλμα λογικής και ονομάζεται σφάλμα σύζευξης (Tversky & Kahneman, 1973). Η προσθήκη λεπτομερειών στο σενάριο σχετικά με την προσωπικότητα, την ακαδημαϊκή εκπαίδευση και τα παλιά ενδιαφέροντα της Λίντα καθιστά τις άλλες επιλογές πιο πειστικές, αλλά λιγότερο πιθανές.			1. The correct answer is Α. Linda is a bank teller 2. The probability of the conjunction of two events, or even three events, cannot be more probable than any one of the events on its own. This is a logical error called the conjunction fallacy (Tversky & Kahneman, 1973). Adding details to the scenario about Linda’s personality, education, and interests makes the other options more believable, but less likely.		
Disease Scenario in Greek			Disease Scenario in English		
Ένας γιατρός δούλευε για αρκετό καιρό πάνω σε μία θεραπεία για μια μυστηριώδη ασθένεια. Τελικά, δημιούργησε ένα φάρμακο που πιστεύει ότι θα θεραπεύσει τους ανθρώπους από τη συγκεκριμένη ασθένεια. Προτού αρχίσει να χορηγεί το φάρμακο, πρέπει να δοκιμάσει την αποτελεσματικότητά του. Επέλεξε 300 άτομα που είχαν την ασθένεια και τους έδωσε το φάρμακο. Ακόμη, επέλεξε 100 άτομα που είχαν την ασθένεια και δεν τους έδωσε το φάρμακο, για να δει τι θα συμβεί. Ο παρακάτω πίνακας δείχνει τα αποτελέσματα του πειράματος:			A doctor had been working on a cure for a mysterious disease. Finally, he created a drug that he thinks will cure people of the disease. Before he can begin to use it regularly, he has to test the drug. He selected 300 people who had the disease and gave them the drug to see what happened. He selected 100 people who had the disease and did not give them the drug in order to see what happened. The table below indicates what the outcome of the experiment was:		
Θεραπεία			Cure		
	Ναι	Όχι		Yes	No
Παρουσία θεραπείας	200	100	Treatment present	200	100
Απουσία θεραπείας	75	25	Treatment absent	75	25
Σύμφωνα με τον πίνακα, το φάρμακο συνδέεται θετικά ή αρνητικά με τη θεραπεία αυτής της ασθένειας; Υποδείξτε την απάντησή σας έχοντας στο νου σας μία κλίμακα το -10 (ισχυρή αρνητική σχέση) έως + 10 (ισχυρή θετική σχέση). Α. -10 έως -1 Β. 0 Γ. +1 έως +10.			According to table, this treatment was positively or negatively associated with the cure for this disease?. Circling a number from a scale ranging from −10 (strong negative association) to +10 (strong positive association). Α. -10 to -1 Β. 0 Γ. +1 to +10		
Feedback in Greek			Feedback in English		
1. Η σωστή απάντηση είναι το A. -10 έως -1 2. Το συγκεκριμένο πρόβλημα είναι ένα πρόβλημα ανίχνευσης συνδιακύμανσης. Η αναλογία θετικών προς αρνητικών αποτελεσμάτων στη θεραπεία της ασθένειας είναι υψηλότερη στη συνθήκη όπου απουσιάζει το φάρμακο σε σύγκριση με τη συνθήκη στην οποία δόθηκε το φάρμακο στους ασθενείς. Στη συνθήκη που δόθηκε το φάρμακο το 1/3 των ασθενών δεν θεραπεύτηκε (100 ασθενείς στους 300). Ενώ, οι 75 από τους 100 ασθενείς που δεν έλαβαν το φάρμακο θεραπεύτηκαν (δηλαδή τα 3/4 των ασθενών). Αν, λοιπόν, συγκρίνουμε αυτά τα δυο αποτελέσματα, καταλαβαίνουμε ότι όχι μόνο το φάρμακο δεν είναι αποτελεσματικό, αλλά έχει αρνητική συσχέτιση με το θεραπευτικό αποτέλεσμα.			1. The correct answer is A. -10 to -1 2. This problem is a covariance detection problem. The ratio of positive to negative outcomes in the treatment of the disease is higher in the condition where the drug was not given than in the condition where the drug was given to the patients. In the condition where the drug was given, 1/3 of the patients were not cured (100 patients out of 300). On the other hand, 75 out of 100 patients who did not receive the drug were cured (i.e. 3/4 of the patients). If we compare these two results, we can see that not only is the drug is not only ineffective, but also negatively correlated with the outcome of the treatment.		

(Kahneman, 2011; Toplak et al., 2011)
